# Reporting Vaccine Complications: What Do Obstetricians and Gynecologists Know About the Vaccine Adverse Event Reporting System?

**DOI:** 10.1155/2013/285257

**Published:** 2013-09-08

**Authors:** L. O. Eckert, B. L. Anderson, B. Gonik, J. Schulkin

**Affiliations:** ^1^Department of Obstetrics & Gynecology, University of Washington, P.O. Box 359865, Seattle, WA 98104, USA; ^2^American College of Obstetricians and Gynecologists, Department of Research, 409 12th Street SW, Washington, DC 20024, USA; ^3^Department of Obstetrics and Gynecology, Division of Maternal-Fetal Medicine, Wayne State University School of Medicine, 3990 John R Street, 7 Brush North, P.O. Box 163, Detroit, MI 48201, USA

## Abstract

*Background*. Obstetrician-gynecologists are increasingly called upon to be vaccinators as an essential part of a woman's primary and preventive health care. Despite the established safety of vaccines, vaccine adverse events may occur. A national Vaccine Adverse Event Reporting System (VAERS) is a well-established mechanism to track adverse events. However, we hypothesized that many obstetrician-gynecologists are naive to the role and use of VAERS. *Methods*. We devised a ten-question survey to a sample of ACOG fellows to assess their knowledge and understanding of VAERS. We performed descriptive and frequency analysis for each of the questions and used one-way analysis of variance for continuous and chi-squared for categorical variables. *Results*. Of the 1000 fellows who received the survey, 377 responded. Only one respondent answered all nine knowledge questions correctly, and 9.2% of physicians had used VAERS. Older physicians were less familiar with VAERS in general and with the specific objectives of VAERS in particular (*χ* ^2^ = 10.7, *P* = .005). *Conclusions*. Obstetrician-gynecologist familiarity with VAERS is lacking. Only when the obstetrician-gynecologist is completely knowledgeable regarding standard vaccine practices, including the availability and use of programs such as VAERS, will providers be functioning as competent and complete vaccinators.

## 1. Introduction

Vaccination against vaccine-preventable diseases is an essential component of women's primary and preventive health care. To provide the best care for our patients, obstetrician-gynecologists are increasingly called to be vaccinators. The indications and types of vaccines recommended for our patients are expanding [1]. Hence, the possibility of adverse events will also increase. A system is in place in this country to track adverse events following vaccine administration: the Vaccine Adverse Event Reporting System (VAERS). VAERS is a voluntary reporting system coadministered by the Centers for Disease Control and the Food and Drug Administration. Established in 1990, VAERS monitors vaccine safety and accepts adverse event (AE) reports following receipt of any US licensed vaccine. However, because reporting to VAERS is not specifically mandated, utilization of VAERS is necessarily dependent on familiarity with the existence of this system.

We hypothesized that experience and familiarity with VAERS are not common among obstetrician-gynecologists. To test this hypothesis and determine more specific knowledge deficits, we conducted a survey to assess the familiarity of practicing obstetrician-gynecologists with VAERS. In this paper, we report the findings of this ten-question assessment.

## 2. Methods

### 2.1. Sample

We invited a total of 1,000 practicing ob-gyns who are members of the American College of Obstetricians and Gynecologists (ACOG) to participate. Three hundred recipients were members of the Collaborative Ambulatory Research network (CARN), a group of physicians who have agreed to participate in 3 to 4 research surveys per year [2, 3]. The remaining 700 were randomly sampled Fellows and Junior Fellows of ACOG. These 700 were chosen by systematically dividing the membership into subgroups of 100 that did not differ in distribution of age, gender, and geographic region and randomly selected 7 groups to participate in this study. The first mailing was sent in September 2011 to all 1,000. Those who did not respond were sent a reminder mailing. A total of three reminder mailings were sent. After the last reminder mailing, a brief letter was sent to nonresponders that included three of the survey questions. The purpose of the letter was to assess whether there were differences between responders and nonresponders on key questions.

### 2.2. Survey and Letter

The survey included the following demographic questions: gender, year of birth, year completed training, state of practice, location of practice, practice setting category, primary medical specialty, race/ethnicity, and primary race/ethnicity of patients. A total of ten questions were asked, nine knowledge questions and one question about the familiarity with VAERS. Question 1 asked about physicians' familiarity with VAERS (in terms of prior use), and Question 2 asked physicians to indicate what the purpose of VAERS is. Questions 3, 4, and 5 asked responders to indicate who sponsors VAERS, the primary objectives of VAERS, and who can report to VAERS. Questions 6 and 7 asked respondents to indicate if they are legally obligated to report an adverse vaccine event to VAERS and what can be reported to VAERS. Question 8 asked physicians to indicate which of seven listed possibilities are recognized limitations of VAERS. Question 9 asked whether the adverse event rate for a vaccine can be calculated using VAERS. The final question asked physicians to select which options were true regarding followup after a report is filed with VAERS. A copy of the survey and the correct answers is included in the Survey below. To examine whether those who had responded to the survey might be more knowledgeable or interested in VAERS than those who did not, we sent a letter with three of the study questions to all of the survey recipients who had not returned a survey. The letter included year of birth, gender, and Questions 1, 7, and 8.

*VAERS Survey *

1. Please give an overall assessment of your familiarity with the Vaccine Adverse Event Reporting System (VAERS) (Please check one):
  - Have used VAERS before (9.2%)
  - Have not used VAERS, but am familiar with its purpose (73.7%)
  - Have not heard of VAERS (17.1%)
2. What is the Vaccine Adverse Event Reporting System (VAERS)? (Please check one):
  - *Post marketing safety surveillance program* (90.5%)
  - Online registry developed by anti-vaccine activists (0.9%)
  - Pre-licensure adverse event reporting system (2.4%)
  - Adverse event compensation program (3.7%)
3. Who sponsors VAERS? (Please select all that apply):
  - *CDC (46.2%)*
  - NIH (13.5%)
  - *FDA (46.5%)*
  - Private industry (21.7%)
4. What are the primary objectives of VAERS? (Please select all that apply):
  - *Detect unusual or rare vaccine adverse events (86.5%)*
  - *Monitor increases in known adverse events related to a vaccine (67.9%)*
  - *Identify patient risk factors for particular types of injuries (46.8%)*
  - *Identify vaccine lots associated with increased reported adverse events (65.1%)*
5. Who can report to VAERS? (Please select all that apply):
  - *Physicians (97%)*
  - *Nurses (81%)*
  - *Pharmacists (62%)*
  - *Vaccine recipient (62%)*
  - *Manufacturers (58%)*
  - *Vaccine recipient spouse (28%)*
6. True/False: If an adverse event occurs with a vaccine, the physician is legally required to report this adverse event to VAERS? (Please check one): 5.2% did not answer
  - True (34.3%)
  - *False (60.6%)*
7. Which is the best statement regarding what can be reported to VAERS? (Please check one):
  - *Any adverse event (65%)*
  - Only those adverse events suspected to be vaccine-related (31.2%)
  - Only those adverse events that require medical attention (2.4%)
  - Only those adverse events that require hospitalization (.3%)
8. What are recognized limitations to VAERS data? (Please select all that apply):
  - *Dose not determine causality (66.0%)*
  - *Underreporting of adverse events (84.4%)*
  - *Increased reporting in the first few years after vaccine licensure (38.4%)*
  - *Increased reporting after documentation of a known or alleged injury (41.9%)*
  - *Reporting of coincidental events (63.2%)*
  - *Data is not complete or necessarily accurate (64.1%)*
  - *Cannot calculate rates of adverse events (40.3%)*
9. True/False: One can calculate the adverse event rate for a vaccine using VAERS? (Please check one): 6.7% did not answer
  - True (21%)
  - *False (72.8%)*
10. After a report is filed with VAERS, which of the following are true? (Please select all that apply):
  - No follow up is available (5.8%)
  - *VAERS staff may request additional information (85.3%)*
  - *Patient consent is not required for release of medical records (20.5%)*
  - Medical records sent to VAERS become public record documents (6.1%).

 Note that correct answers are in italic with (%) of responders who chose each option.

### 2.3. Data Analysis

We computed descriptive statistics for all questions. For nine of the ten questions, participants were asked knowledge questions that have correct answers. For these questions, participants were grouped into two groups (answered correctly or answered incorrectly), and we computed the percent of respondents answering correctly. We assessed differences in age and gender between those who answered the question correctly and those who did not. For questions without a correct answer (which include only one question about familiarity with VAERS), we analyzed differences in age and gender among the response options.

In obstetrician-gynecologists, age and gender are highly associated. Hence, when gender differences were assessed, we controlled age using a dichotomous variable. Participants were grouped using a median split into two roughly equal sized groups: physicians born between 1933 and 1958 (51%, *N* = 188/372) and physicians born between 1959 and 1979 (49%, *N* = 184/372). Separate tests for differences between males and females were run for the older age group and the younger age group. We reported only significant differences.

Data were analyzed using a personal computer based version of SPSS 17.0 (SPSS Inc., Chicago, IL). One-way analysis of variance was used for continuous variables; *χ* ^2^ analyses were conducted for categorical variables. Significance was evaluated at *α* = 0.05. 

## 3. Results

The response rate for CARN participants was 57% (171/300). For non-CARN participants, the response rate was 32.1% (225/700). Overall, a total of 397 physicians responded to the survey (39.7% response rate). One responder could not be identified as CARN or non-CARN. Twenty responders were considered ineligible and were eliminated from data analysis (i.e., 9 physicians indicated being retired, and 11 physicians returned the survey blank), leaving 377 eligible responders (167 CARN, 209 non-CARN, and one unidentified). Of the 377 eligible responders, 50 did not complete the survey and were excluded from analysis, resulting in a total sample of 327 obstetrician-gynecologists. We found no significant differences between CARN and non-CARN responders (data not shown); therefore, we collapsed the data and analyzed in aggregate form. 

### 3.1. Demographics

The sample demographics are presented in Table 1. Average year of birth was 1958 (±9.7), and male physicians (1954 ± 9.4) were significantly older than female physicians (1962 ± 8.3) (*F*(1, 318) = 67.3, *P* < .001). White, non-Hispanic was the most common race of physicians (82%) and their patients (72%). Around half (54%) were in group practice and 73% practice general obstetrics and gynecology.

### 3.2. VAERS Survey Questions

The provided VAERS Survey shows the responses to all of the survey questions. Overall, only one respondent answered all nine knowledge questions correctly. When asked about the sponsors of VAERS, only 12.5% correctly indicated that the CDC and FDA are the sponsors and that NIH and private industry are not. A total of 43.4% correctly selected all four objectives of VAERS. A total of 24.2% of the sample correctly indicated all six of the individuals listed can report to VAERS. When asked what happens after a report is filed, 17.4% correctly indicated that VAERS staff could request additional information and that patient consent is not required. 

The number of recognized limitations to VAERS identified by each physician was summed for a total “limitations score.” The mean “limitations score” was 4.0 (SD = 2.0). Differences in age were found among females only; older female physicians had a higher mean limitations score (*M* = 4.6, SD = 2.0) than younger female physicians (*M* = 3.9, SD = 2.1) (*F*(1, 161) = 4.4, *P* = .038).

**Figure 1:**
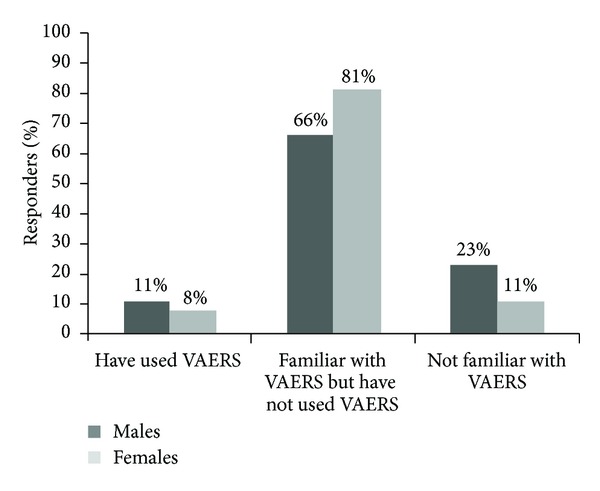
Familiarity with VAERS broken down by males and females (data not controlled for age) (*χ* ^2^ = 10.7, *P* = .005) (chi-square test for overall differences).

**Figure 2:**
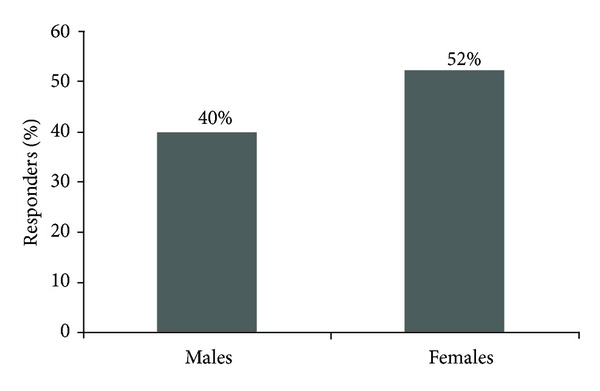
Percent of males and females that know all of the objectives of VAERS (data not controlled for age) (*χ* ^2^ = 4.2, *P* = .041).

Gender differences were found on some of the survey questions. As shown in Figure 1, males were twice as likely to be “not familiar” with VAERS than females, with 23% of males indicating “not familiar” compared with 11% of females (*χ* ^2^ = 10.7, *P* = .005). When assessing for differences in gender among younger and older physicians, males were more likely to indicate “not familiar” than females in the older age group (*χ* ^2^ = 8.6, *P* = .014), but not in the younger age group. As shown in Figure 2, females were more likely than males to indicate correctly all four objectives of VAERS (40% of males versus 52% of females, *χ* ^2^ = 4.2, *P* = .041). When assessing for differences in gender among younger and older physicians, females were more likely to indicate the correct VAERS objectives in the older group (39% of males versus 61% of females, *χ* ^2^ = 6.5, *P* = .01), but there was no significant difference between males and females in the younger group. 

### 3.3. Letter Responses and Comparison with Survey Responders

A total of 77 ob-gyns returned a letter. Of the 77, 12 were retired or did not complete over half of the questions on the letter and were therefore excluded. Therefore, a total of 65 letter responders were included in the letter analysis.

We compared the survey responses with letter responses to assess potential differences between those who responded to the survey and those who did not respond. The number of eligible letter responders was 65, with 18 from the CARN group and 47 from the non-CARN group. Letter responders were not significantly different from survey responders in gender or year of birth. The mean year of birth was 1960 (SD = 8.6). Forty-three percent were female, 46.2% were male, and 10.8% did not identify their gender. We found no significant differences between survey responders and letter responders on any of these questions. This suggests that the answers we received to the survey are similar to those we would have received even if we had had a larger response rate. 

## 4. Discussion

Safety concerns are one of the most common immunization concerns cited by patients [4]. In fact, because of potential impact on the fetus, in our specialty, safety concerns can be amplified both for patients and providers for immunizations recommend during pregnancy [5–7]. We have seen that data generated from VAERS is able to reinforce the safety profile for recommending vaccines during pregnancy, including H1N1 and Tdap [8–10]. With increased reporting to VAERS, the data base will become more robust. Currently, most obstetrician-gynecologists acknowledge familiarity with VAERS. However, only one respondent correctly answered all the questions correctly and few (9%) obstetrician-gynecologists surveyed have used VAERS. Hence, we verified our original hypothesis that there is a lack of familiarity with VAERS in the ob/gyn's professional community.

To determine more specific information about knowledge deficits, we stratified the answers to this survey based on age and gender. When we compared age differences, older physicians were less familiar with VAERS in general and with the specific objectives of VAERS in particular. For controlling age, we stratified the data by gender and found that women were more likely than men to state that they were aware of VAERS and also to know all the objectives of VAERS. When stratifying both by age and gender, older women were more likely to know the correct answers to VAERS objectives than older males, but in the younger group, the gender differences were not seen. This study does not address attitudinal or practice differences in immunization administration nor were we able to find other studies documenting the frequency of immunization administration in obstetrician-gynecologist practices based on provider age or gender. However, studying these differences could provide useful insight into how we see ourselves or practice as providers of immunizations and allow more targeted CME strategies. 

This survey addresses knowledge and use of VAERS. As a survey with a response rate of 37% and a sample size of 1000, this study has some weakness in generalizability. Those who responded to the survey may be more likely to have knowledge or interest in VAERS. We tested this by sending all nonresponders a brief letter soliciting responses to three of the survey questions. When comparing survey responders and letter responders, we did not see differences in familiarity with VAERS and prior use of VAERS supporting the generalizability of our survey. While some differences may be present between responders and nonresponders, these differences may not be as important as the similar low prior use of VAERS between these two groups. 

Regarding obstetrician-gynecologist use and knowledge of VAERS, the message is simple and clear: it could be better. More broadly, the role of ob/gyns as immunizers also could improve. We can be encouraged that about 50% of eligible pregnant patients received the flu vaccine in 2011 (compared to 17% prior to the H1N1 pandemic). However, we can utilize a multifactorial approach to further improve the knowledge about and integration of immunizations into the ob-gyn's practice. The American Congress of Obstetricians and Gynecologists (ACOG) has launched a website, which provides scientific and practical information to facilitate integration of immunizations into clinicians' offices [1]. Also, CME courses are commonly offered at ACOG's annual clinical meeting for immunizations. Modules on immunization for maintenance of certification have been added. Residents' education on immunizations is now a requirement [11]. In areas of the country where a vaccine preventable epidemic has occurred, such as the recent epidemic of pertussis in the state of Washington [12], pregnant patients have been directly targeted and encouraged to solicit immunizations from their providers. Adding immunization questions to written and oral board specialty certification examinations as well as the written recertification examination would also necessitate an immunization fund of knowledge for practicing ob-gyns. Specific to VAERS, Haber and colleagues [13] have recently demonstrated the value of internet-based reporting as a means to improve provider utilization of this safety monitoring system. 

The medical benefits of immunizations are clear. The benefits to our patients of increasing vaccine coverage are also clear. This survey demonstrates that familiarity, understanding, and use of the VAERS data base merit improvement. Further research comparing attitudes and practice patterns of obstetrician-gynecologists regarding immunization is also merited so that we can strategically implement education efforts to enhance obstetrician-gynecologist's utilization of vaccines: a proven primary prevention tool.

## Figures and Tables

**Table 1:** Demographic variables of responding physicians (N = 327).

Demographic variables	*N* (%) or mean (SD)
Age (year of birth)	1958 (±9.7)
Females	1962 (±8.3)
Males	1954 (±9.4)
Gender	
Male	153 (47%)
Female	171 (53%)
Practice location	
Urban-inner city	148 (45%)
Suburban	126 (39%)
Other	48 (15%)
Practice type	
Solo private practice	63 (19%)
Group practice	177 (54%)
Community hospital facility	35 (11%)
University full-time faculty and practice	31 (10%)
Other (i.e., public health, government, volunteer, etc.)	19 (6%)
Primary medical specialty	
General obstetrics and gynecology	238 (73%)
Gynecology only	66 (20%)
Obstetrics only	6 (2%)
Other (i.e., REI, urogynecology, etc.)	15 (5%)
Race/ethnicity	
White, non-Hispanic	268 (82%)
White, Hispanic	13 (4%)
Asian/Pacific Islander	28 (9%)
African American	10 (3%)
Native American/multiracial	6 (2%)
Primary race/ethnicity of patients	
White, non-Hispanic	233 (72%)
White, Hispanic	27 (8%)
African American	21 (6%)
Multiracial	34 (10%)
Asian/Pacific Islander/native American/unsure	10 (4%)
